# ^18^F-FDG PET/CT Imaging: Normal Variants, Pitfalls, and Artifacts Musculoskeletal, Infection, and Inflammation

**DOI:** 10.3389/fnume.2022.847810

**Published:** 2022-03-21

**Authors:** Olwethu Mbakaza, Mboyo-Di-Tamba Willy Vangu

**Affiliations:** Department of Nuclear Medicine and Molecular Imaging, Charlotte Maxeke Johannesburg Academic Hospital, University of the Witwatersrand, Johannesburg, South Africa

**Keywords:** ^18^F-FDG PET/CT, musculoskeletal, pitfalls, artifacts, normal variants, inflammation, infection

## Abstract

^18^F-FDG PET/CT is an integral part of modern-day practice, especially in the management of individuals presenting with malignant processes. The use of this novel imaging modality in oncology has been rapidly evolving. However, due to its detection of cellular metabolism, it is not truly tumor specific. ^18^F-FDG is also used in the detection of infective and inflammatory disorders. One of the challenges experienced with ^18^F-FDG PET/CT imaging is the correct differentiation of abnormal uptake that is potentially pathologic, from physiological uptake. Imaging readers, particularly the nuclear physicians, therefore need to be aware of normal physiological variants of uptake, as well as potential pitfalls and artifacts when imaging with ^18^F-FDG. This is true for musculoskeletal uptake, where more than often, infective and inflammatory processes should not be mistaken for malignancy. This article aims to provide a pictorial review and analysis of cases that depict musculoskeletal, infective, and inflammatory uptake as normal variants, pitfalls, and artifacts on ^18^F-FDG PET/CT imaging. The impact of this article is to help in the minimizing of poor imaging quality, erroneous interpretations and diminishes misdiagnoses that may impact on the adequate management of patients with undesirable consequences.

## Introduction

Flourine-18 fluorodeoxyglucose positron emission tomography/computed tomography (^18^F-FDG PET/CT) is today widely used in the management of oncology patients. Due to its non-specific nature for malignant lesions, ^18^F-FDG may also be used to detect processes involved in infection and inflammation. Therefore, issues related to pitfalls and normal variants should be kept in mind during imaging interpretation. This article will be focusing on imaging illustration pitfalls and normal variants related to musculoskeletal, infection, and inflammation with limited narrative related to the topic. Broader narrative on normal variants, pitfalls, and artifacts for ^18^F-FDG PET/CT in general may be found in other published articles.

### FDG Physiology

FDG is a glucose analog and thus follows the similar fate as glucose in living tissues. Glucose enters the cell through glucose transporters, gets phosphorylated to glucose-6 phosphate by hexokinase, and further metabolism occurs (1). FDG, however, does not get metabolized further once it is phosphorylated to glucose-6 phosphate and is trapped inside the cell. FDG uptake inside the cells is dependent on glucose transporters, which indirectly get affected by serum glucose level, insulin level, and cellular demand (1).

The quantitative measurement of ^18^F-FDG accumulation in tissues has not yet been standardized (2). The commonly used parameter for quantitative measurement is the maximum standardized uptake value (SUVmax). Related quantitative parameters include, among others, the SUVpeak, SUVmean, total lesion glycolysis (TLG), and metabolic tumor volume (MTV) (2).

### Pathophysiology of ^18^F-FDG in Malignancy

^18^F-FDG PET/CT plays a pivotal role in imaging malignant processes, and has been used to detect and evaluate both solid and haematological maligancies (3). FDG, as a glucose analog, adds a benefit of depicting functional information—hence metabolic abnormalities before anatomic changes occur (4). This is based on increased glucose uptake and increased glycolytic activity in malignant cells (3).

^18^F-FDG PET/CT is a very useful tool in the diagnosis and follow-up of malignant processes, due to the sensitivity of the tracer and imaging modality. It aids in upstaging and downstaging of the disease extent, which leads to a change in disease management (5). ^18^F-FDG PET/CT is useful in the detection of malignant disease with high sensitivity; it does, however, have the limitation of low specificity—in that a highly metabolically active lesion may be indicative of malignant, reactive, or reparative changes, and infective or inflammatory changes (1). To increase its specificity, it may be of importance to review the patient's clinical history, particularly oncologic history, such as treatment and procedures. This is in addition to other imaging modalities, together with laboratory changes (3). This clinical information may be in a prepared questionnaire or obtained in person. Despite these measures, there will be lesions with indeterminate uptake of tracer on ^18^F-FDG PET/CT (3).

### Biodistribution of FDG

The physiological biodistribution of FDG is the brain (gray matter), vocal cords, tonsils, thymus, skeletal muscle, brown fat, left ventricular myocardium, bowel, liver, spleen, kidneys, ureter, urinary bladder, and bone marrow (6). Physiological uptake in the gingival, genioglossus, and pterygoid muscles has also been described (7).

Various (musculoskeletal, infective, and inflammatory) processes that may be mistakenly interpreted as malignant changes will be discussed by way of a pictorial review below.

### Musculoskeletal Pitfalls

#### Multiple Sites of Skeletal Trauma

**Figure 1:**
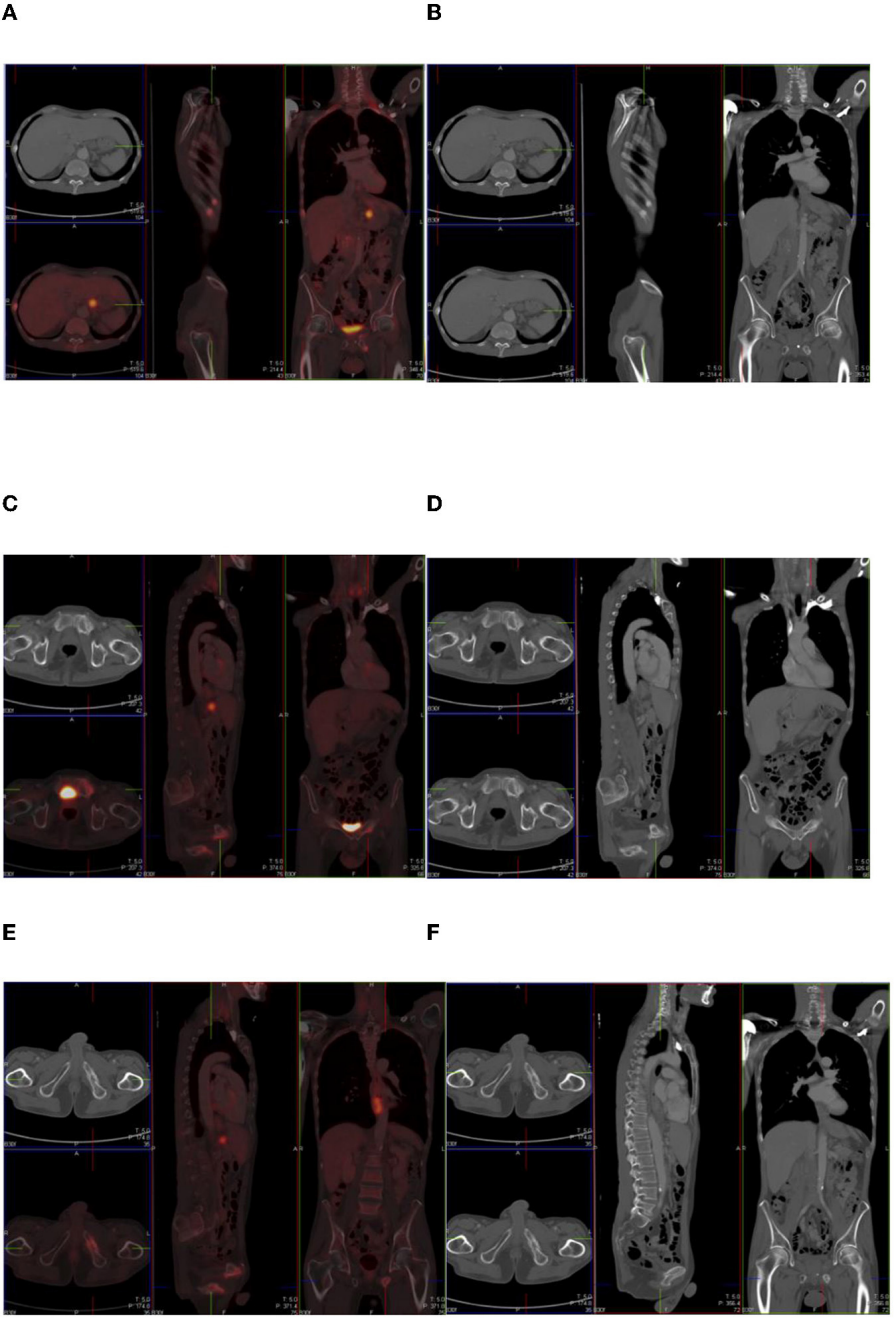
Combined PET/CT and CT images of a 54-year-old man with esophageal melanoma. He has multiple sites of skeletal uptake attributable to recent trauma: in the R lateral 8th and 9th ribs **(A,B)**, superior pubic ramus **(C,D)**, and inferior pubic ramus **(E,F)**.

Bone metastases may be represented by discrete foci of uptake in bone, which is a similar presentation as fractures (7). A fracture line may also be missed if the window displayed is in the CT soft tissue window, as opposed to bone window (6). Patients with underlying malignancy who have received chemotherapy with or without radiation therapy can also present with pelvic insufficiency fractures. Additional risk factors to this include corticosteroid therapy, osteoporosis, rheumatoid arthritis, and metabolic bone disease (7). A thorough history of trauma needs to be ascertained from the patient as per case in Figure 1.

### Inflammatory Pitfalls

#### Pathophysiology of ^18^F-FDG in Inflammation

Inflammation is the tissues' response to injury, which may include irritation, infection, or trauma. The body responds to inflammatory stimuli with a cascade of events, which includes local hyperemia, release of proteins such as fibrin and immunoglobulins, leakage of fluids, and infiltration of inflammatory cells (8). Inflammation exhibits ^18^F-FDG uptake due to the recruitment of activated white blood cells (neutrophils and lymphocytes), which have high affinity for glucose transporters, especially GLUT 1 and GLUT 3 (1, 8). There is also upregulation of GLUT-1 transporters in macrophages, which constitute a major component in the body's response to infection (1). There is also increased affinity to ^18^F-FDG in inflammation through cytokines and growth factors (8). Increased uptake because of infection or inflammation on ^18^F-FDG PET/CT cannot be distinguished from tumor uptake.

**Figure 2:**
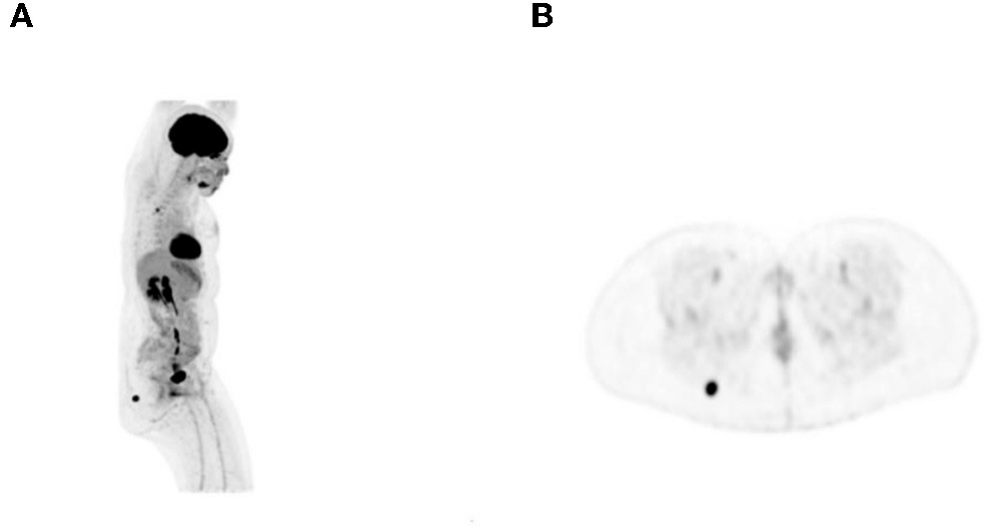
A 42-year-old woman with Hodgkin's lymphoma. ^18^F-FDG PET maximum intensity projection (MIP) **(A)** and transaxial **(B)** images showed focal intense uptake in the right buttock. Fine-needle aspiration cytology showed nodular fasciitis.

**Figure 3:**
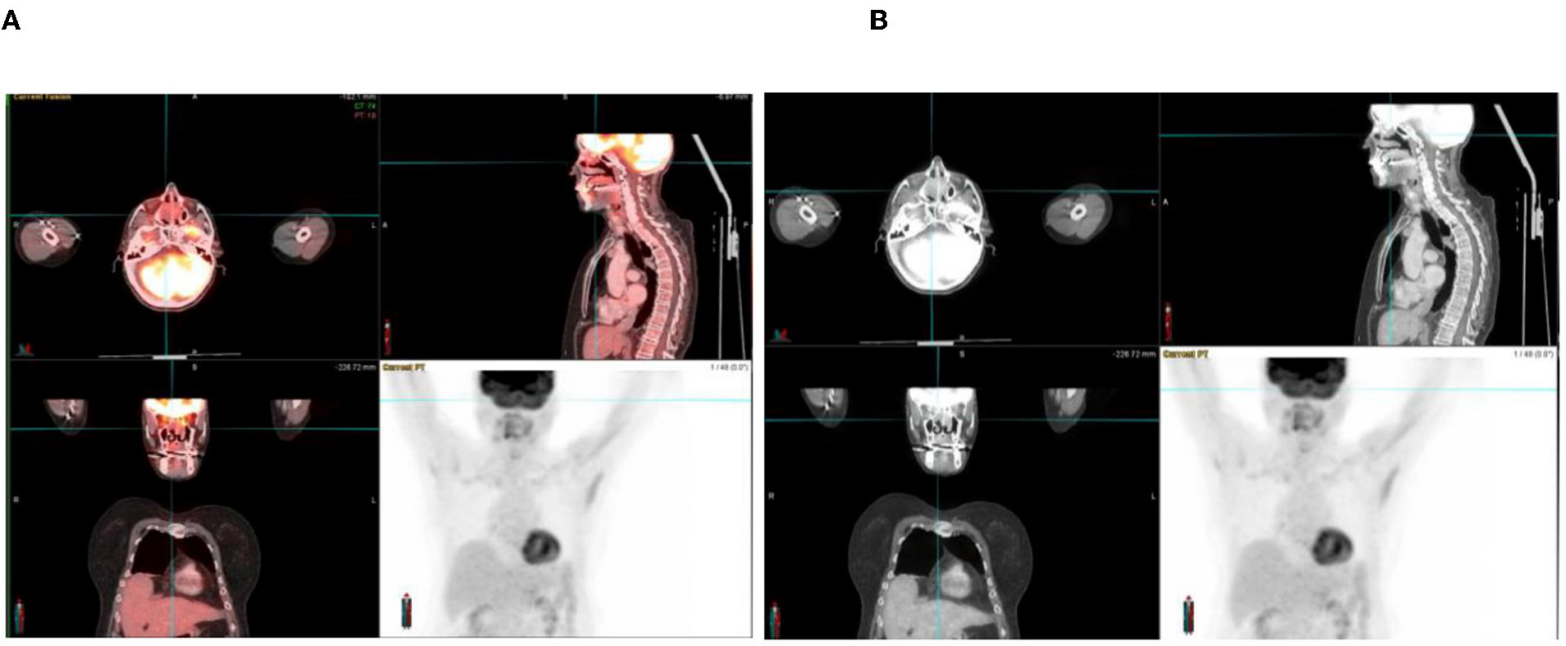
A 70-year-old woman with adenocarcinoma of the colon. ^18^F-FDG PET/CT **(A)** and CT **(B)** images show increased FDG uptake in an opacified nasal passage, which reflects inflammation rather than metastases.

**Figure 4:**
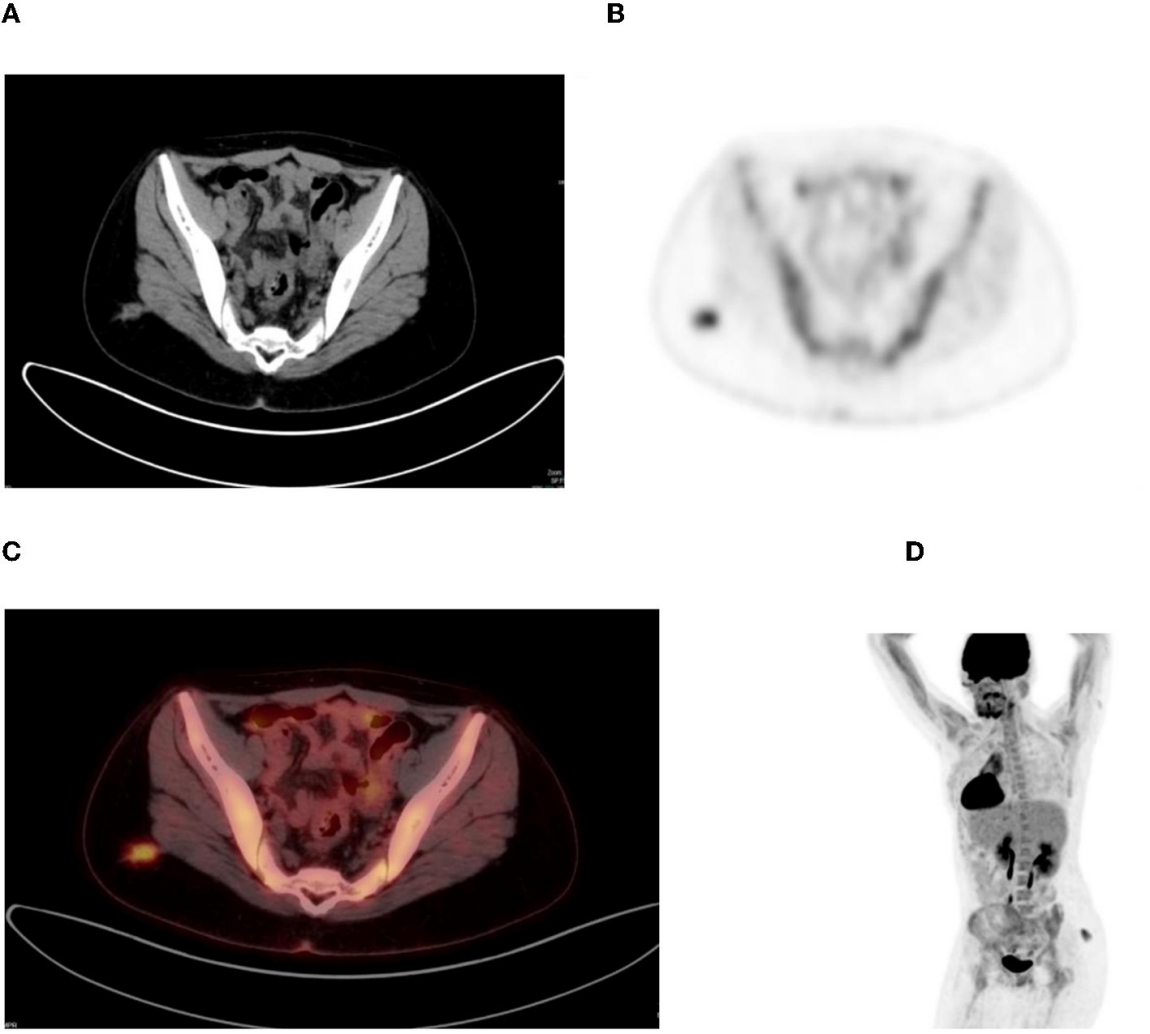
Young woman with Hodgkin's disease and underlying retroviral disease. CT **(A)**, PET **(B)**, combined PET/CT **(C)**, and MIP **(D)** images show focal increased uptake in the right buttock, most likely inflammatory.

Figures 2–4 show patterns of ^18^F-FDG uptake consistent with inflammation rather than malignant disease.

### Infection

**Figure 5:**
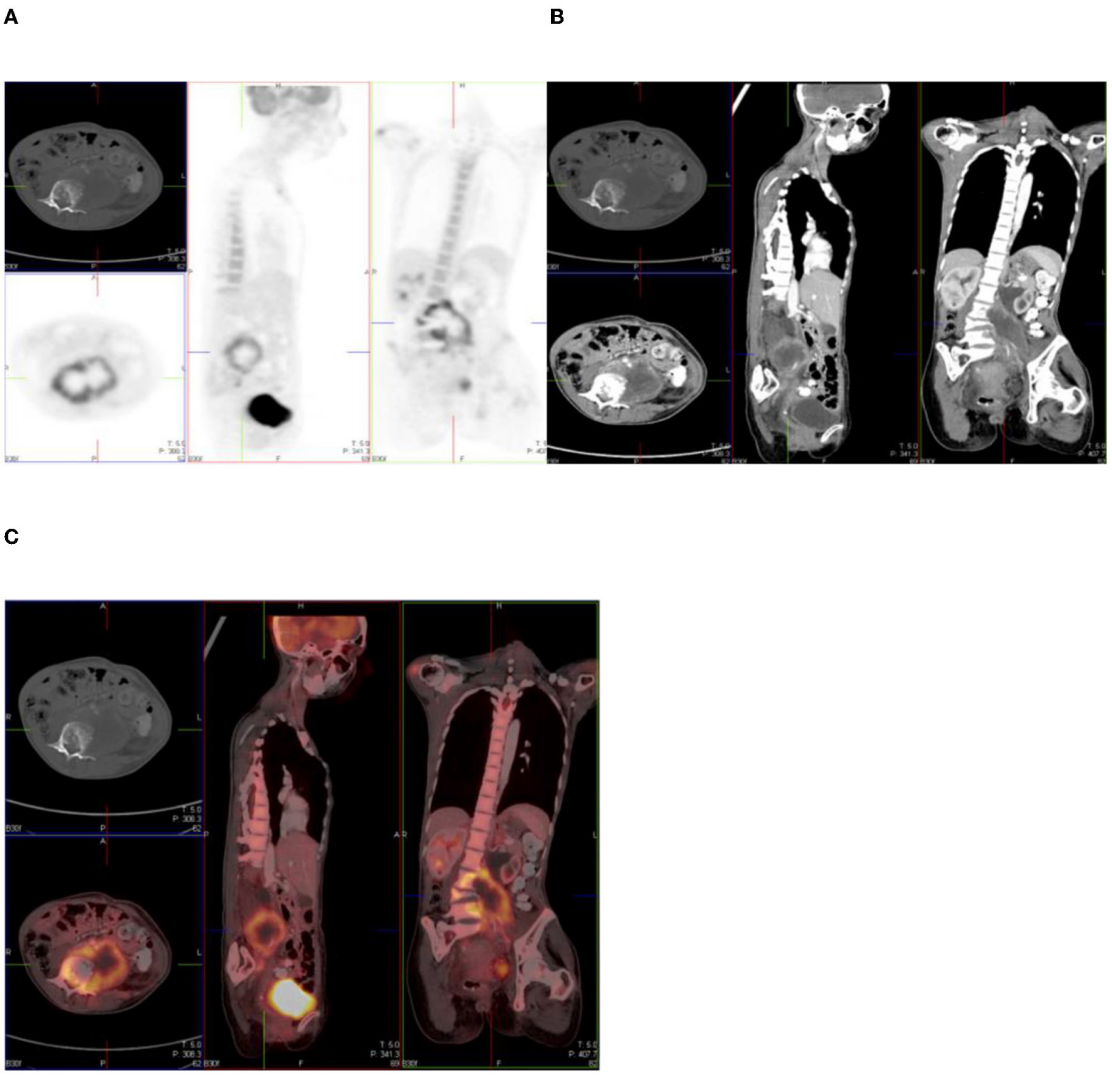
^18^F-FDG PET **(A)**, CT **(B)**, and combined PET/CT **(C)** images of a 38-year-old woman with retroviral disease and stage III Ca cervix. She has a metabolically active collection in the left psoas muscle—with radiological features of a cold abscess which, in this case, is secondary to TB. The left psoas collection extends into the vertebral column with resultant compression fracture of the vertebral body of L4.

**Figure 6:**
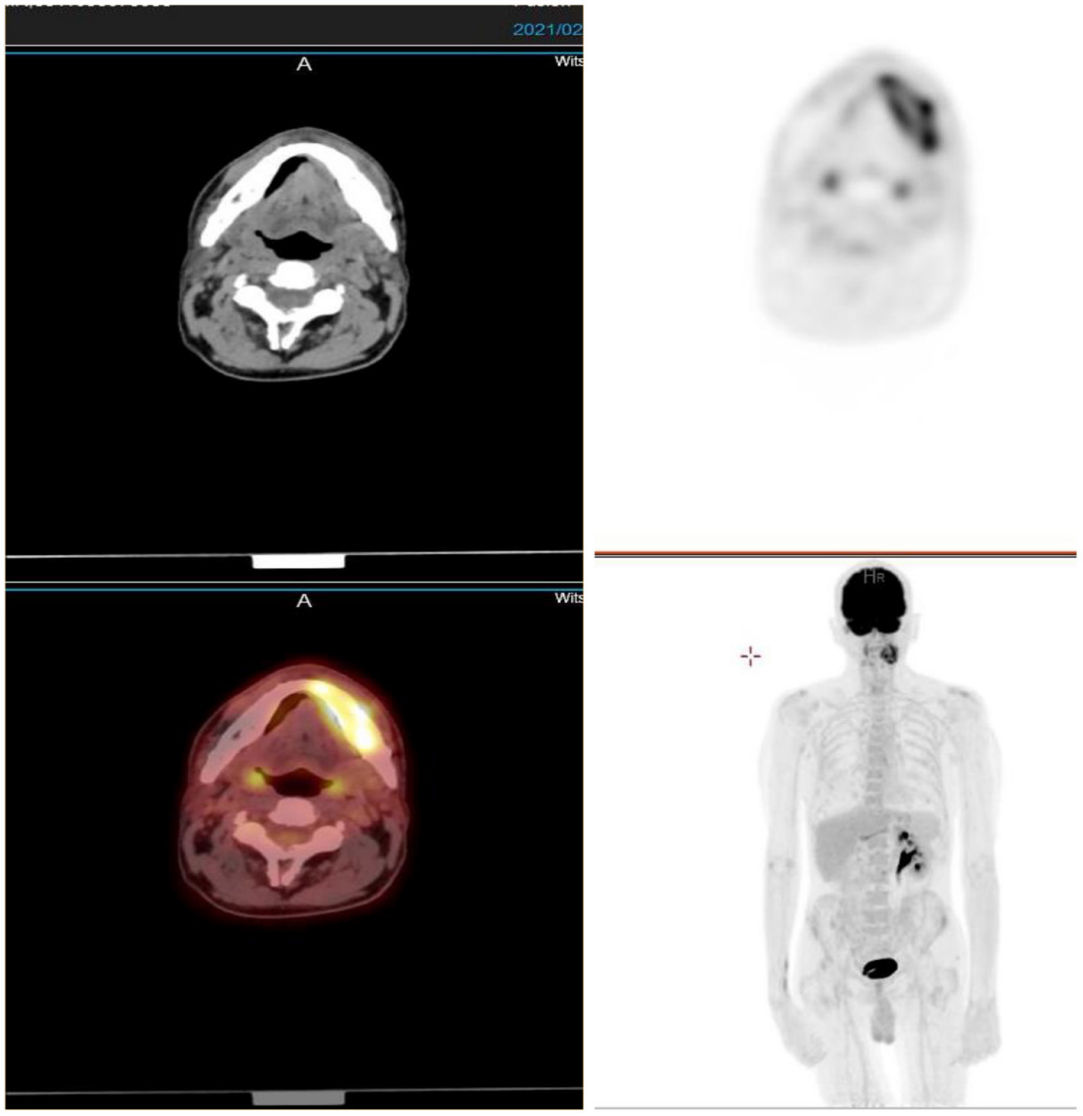
A 54-year-old man with renal cell carcinoma. Combined PET/CT images showed an intense lesion in the left side of the mandible, which was due to osteomyelitis.

Cervical carcinoma is an AIDS-defining illness (9, 10). Immunocompromised women living with HIV with concomitant human papillomavirus infection have a higher chance of developing pre-invasive lesions, which lead to cervical carcinoma (8). ^18^F-FDG PET is of use in the diagnosis, staging, and detection of metastasis and in post-treatment monitoring of several AIDS-defining malignancies (11). However, caution should be taken, as immunosuppressed patients are also prone to infection. Sites of infection may mimic metastatic disease as in the case of the 38-year-old woman with a stage IIIB cervical carcinoma in aforementioned Figure 5. This is a clinical situation of an immunocompromised individual with retroviral disease on antiretroviral treatment. The psoas collection has radiological features of a cold abscess. Osseous infection may also mimick metastatic disease, as in this case of a 54 year old man with renal cell carcinoma. His combined FDG PET/CT images (Figure 6) showed an intense lesion in the left side of the mandible, which was due to osteomyelitis.

### Musculoskeletal Uptake: Normal Variants

The major source of energy for skeletal muscles during the resting state is fatty acid oxidation (1). This results in homogeneous uptake of FDG in skeletal muscle. Plasma insulin, however, can increase glucose uptake in skeletal muscle by inducing the translocation of GLUT-4 from the intracellular vesicles to the plasma membrane and can thus result in increased skeletal glucose uptake in postprandial state (1). Voluntary and involuntary muscular activity can result in increased FDG uptake, which may result in a wide variety of seemingly pathological uptake.

Diffuse whole-body muscle uptake may be seen in patients with recent insulin injection, strenuous exercise involving strenuous muscle groups, and recent meal consumption (1).

Activities such as talking can cause increased uptake in bilateral vocal cords. Muscular uptake involving the upper extremities could result from activities that require the use of skeletal muscle such as turning pages of a book (1).

**Figure 7:**
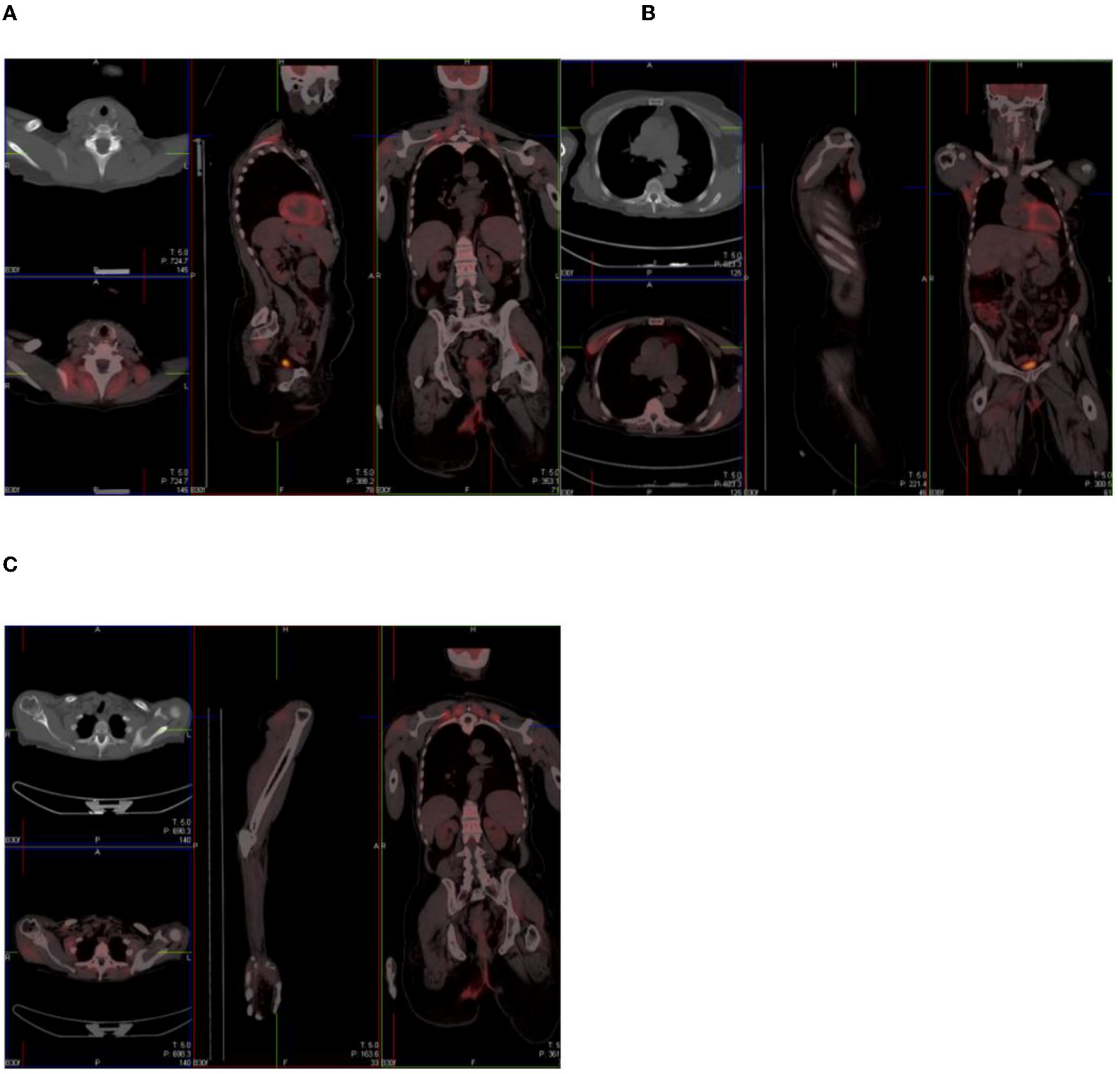
Combined PET/CT images of a 67-year-old woman with anal carcinoma. There is diffuse uptake in the skeletal muscles of the neck **(A)**, chest **(B)**, and upper limbs **(C)**—related to muscular exertion.

In individuals who experience muscle exertion, uptake may be seen in different parts of the body (Figure 7). In the same vein, in individuals with chronic obstructive pulmonary disease, due to difficult breathing and/or excessive coughing, intercostal muscular and diaphragmatic uptake may be seen.

### Osteodegenerative Changes and Arthropathies

**Figure 8:**
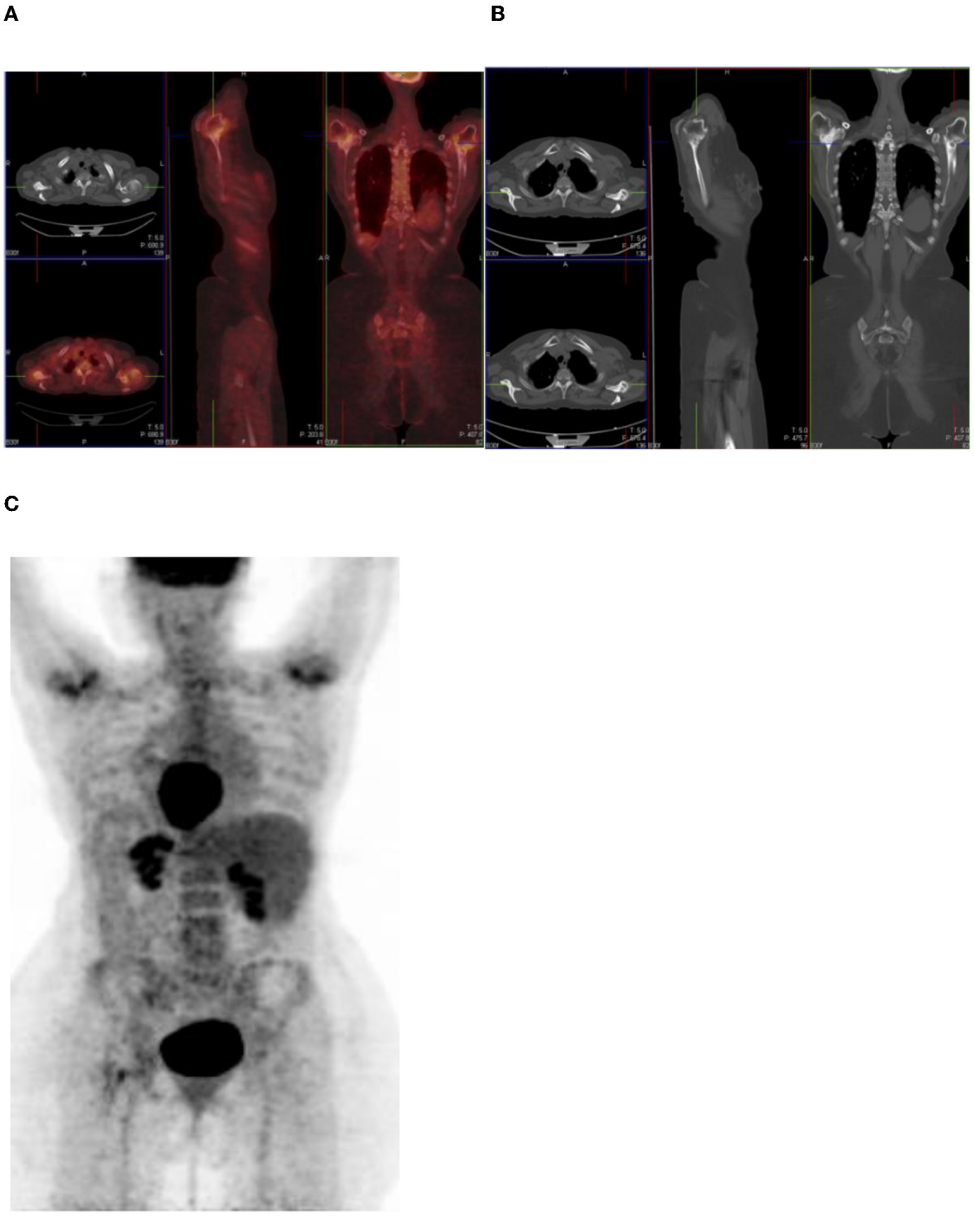
A 69-year-old woman with left breast carcinoma. Combined PET/CT **(A)** and PET maximum intensity projection (MIP) **(C)** images show intense uptake in the gleno-humeral joints, with subchondral cystic osteodegenerative changes on CT **(B)**.

Active inflammatory arthropathies because of psoriasis, rheumatoid arthritis, gout, and ankylosing spondylitis may mimic disease. The uptake of tracer in these conditions is dependent on the presence of synovitis (7). Figure 8 shows an ^18^F-FDG PET/CT study done on a 69-year-old woman with left breast carcinoma, which showed intense uptake in the gleno-humeral joints, with subchondral cystic osteodegenerative changes on CT.

**Figure 9:**
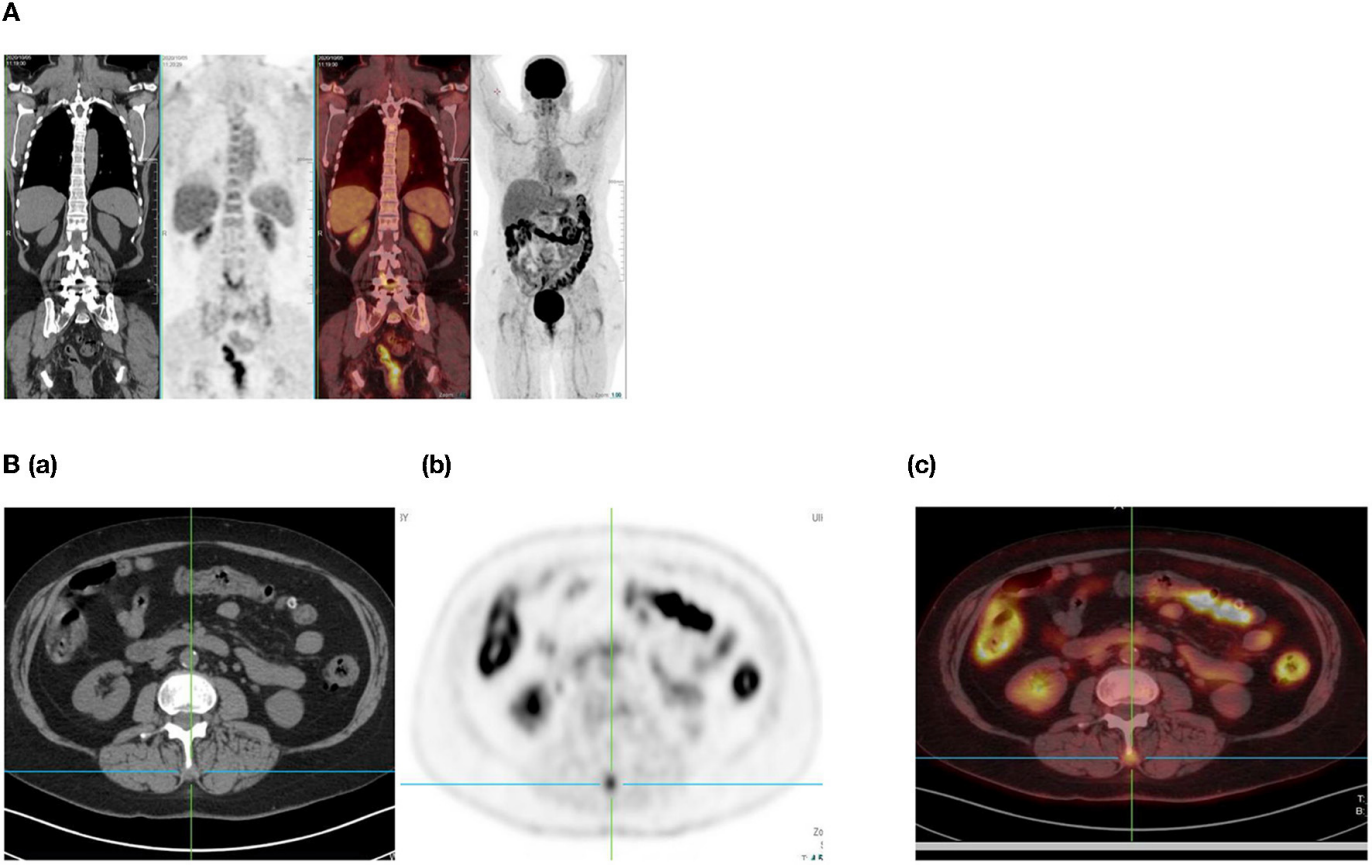
**(A)** A 68-year-old woman with basal cell carcinoma of the left breast. Combined PET/CT images show intense uptake in the right facet joint of L3/L4, which is consistent with osteodegenerative change. **(B**a-c**)** Same patient as in **(A)** above with increased uptake in the spinous process.

**Figure 10:**
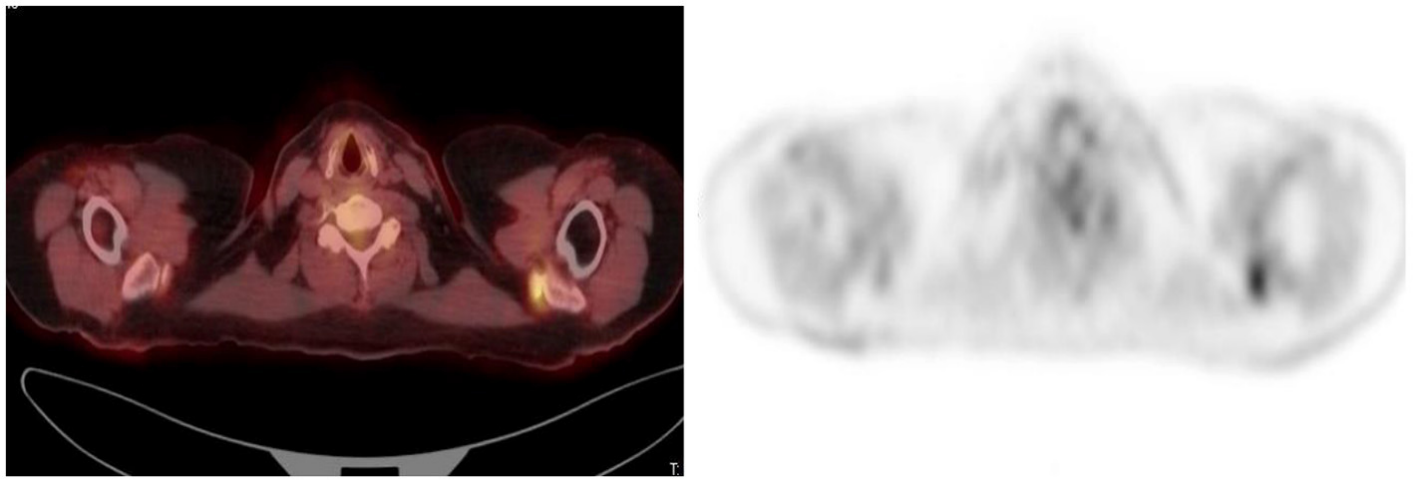
56 year old woman with ovarian Ca. Combined PET/CT and PET images show intense uptake in the left acromio-clavicular joint, which is osteodegenerative.

**Figure 11:**
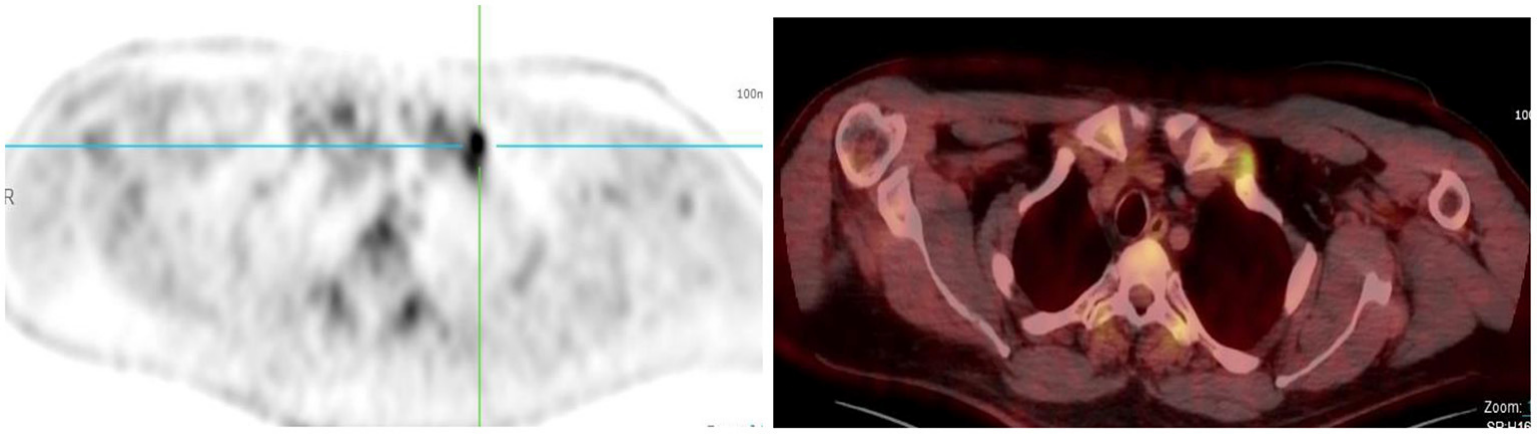
50 year old man with melanoma of the scalp. PET and Combined PET/CT images show intense uptake in the left sternoclavicular joint, which is osteodegenerative.

**Figure 12:**
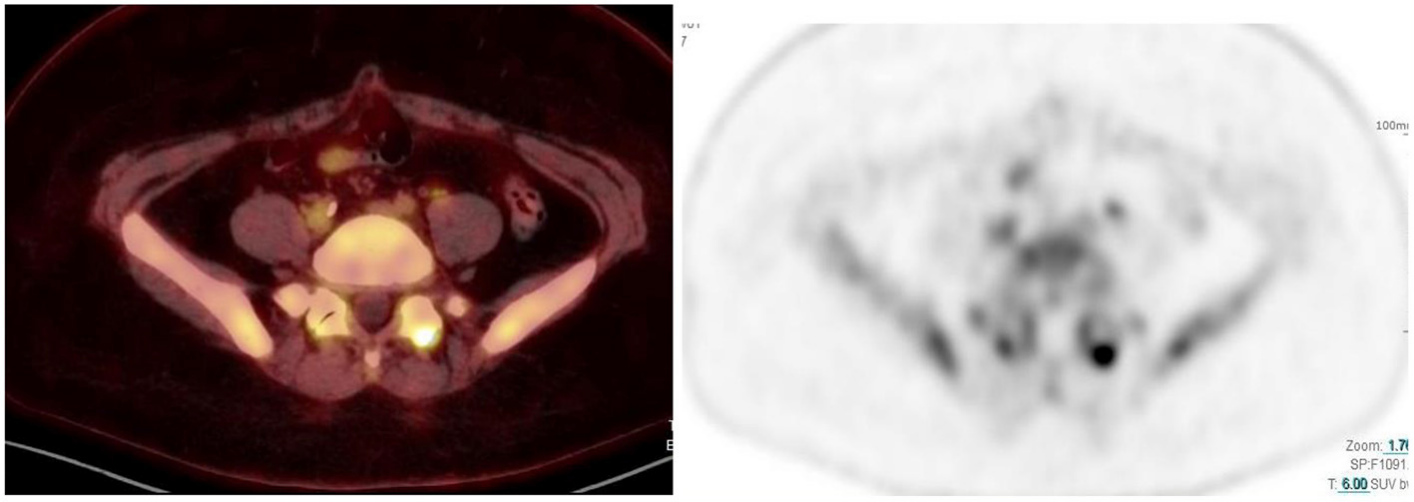
59 year old woman with breast Ca. Combined PET/CT and PET images show intense uptake in the left facet joint of L5/S1, which is osteodegenerative.

A number of images (Figures 9–12) that represent multiple sites of intense FDG activity in the skeleton are displayed below. All changes were due to osteodegenerative changes.

In a study evaluating cervical, thoracic, and lumbar spine uptake in 150 patients who underwent ^18^F-FDG PET/CT scan, Costelloe et al. found abnormal uptake corresponding to osteodegenerative change in 22% of patients (7). They, however, found a weak correlation between severe osteodegenerative changes and the degree of uptake (7).

### Metallic Artifacts

**Figure 13:**
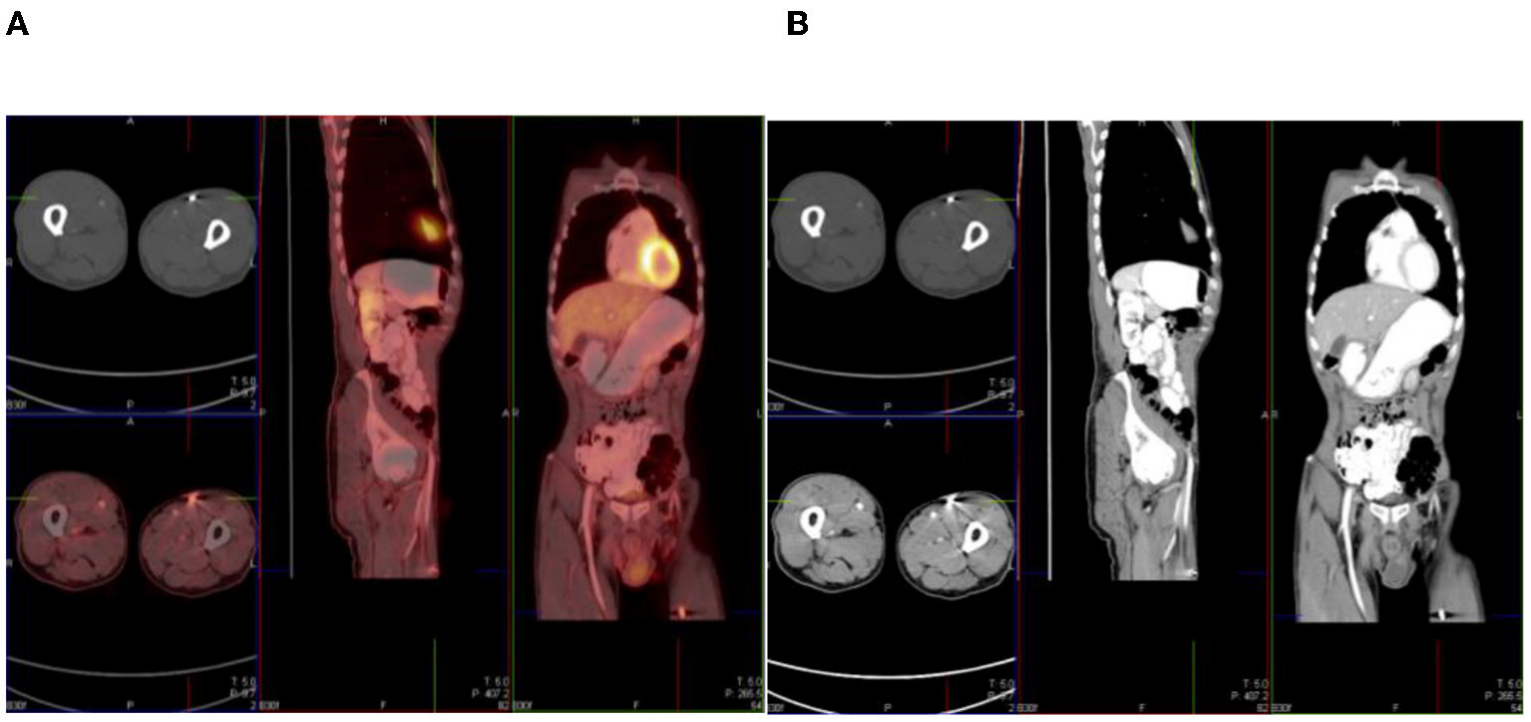
A 56-year-old man with sinonasal carcinoma of the hard palate. Combined PET/CT **(A)** and CT **(B)** images show intense uptake related to a metallic artifact in the left anterior thigh.

Metallic objects such as orthopedic hardware, dental implants, pacemakers, and injection ports attenuate photons, and the degree of attenuation is higher at CT X-ray energy than at PET energy (12). This therefore leads to an overestimation of attenuation, which results in artifactually increased FDG activity in CT attenuation-corrected PET images (12) (Figure 13). If an artifact is suspected, then confirmation is made by evaluating the non–attenuation-corrected images. This is to prevent erroneous misinterpretation of increased uptake as disease.

## Conclusion

^18^F-FDG PET/CT is a very useful tool in the diagnosis and follow-up of malignant disease due to the high sensitivity of this imaging modality. Its specificity, however, may be reduced by the presence of musculoskeletal, infective, and inflammatory pitfalls. It is important to always be wary of these potential pitfalls as they may influence the diagnosis and course of management of the patient. The patient's clinical history is a mandatory step in the navigation of PET/CT images.

## Author Contributions

OM responsible for the data collection, data sources, and write-up of the article. M-D-TV responsible for conceptualization of the topic and write-up and review of the article. Both authors contributed to the article and approved the submitted version.

## Conflict of Interest

The authors declare that the research was conducted in the absence of any commercial or financial relationships that could be construed as a potential conflict of interest.

## Publisher's Note

All claims expressed in this article are solely those of the authors and do not necessarily represent those of their affiliated organizations, or those of the publisher, the editors and the reviewers. Any product that may be evaluated in this article, or claim that may be made by its manufacturer, is not guaranteed or endorsed by the publisher.
